# ‘PePApipe’: A complete bioinformatics analysis pipeline for African Swine Fever Virus genome

**DOI:** 10.1371/journal.pone.0356006

**Published:** 2026-09-10

**Authors:** Vicente Lopez-Chavarrias, Irene Aldea, Jovita Fernández-Pinero

**Affiliations:** Department of Infectious Diseases and Global Health, Centro de Investigación en Sanidad Animal (CISA), Instituto Nacional de Investigación y Tecnología Agraria y Alimentaria (INIA), Consejo Superior de Investigaciones Científicas (CSIC), Valdeolmos, Madrid, Spain; Chattogram Veterinary and Animal Sciences University, BANGLADESH

## Abstract

African Swine Fever Virus (ASFV) is of high concern in porcine livestock across the world due to both the high mortality rates and the trade restrictions imposed on affected regions. The viral genome is large and complex, and genomic analysis is essential for tracing its origin and evolution. Although several bioinformatics tools exist for genome assembly and analysis, no single platform integrates all necessary steps in an accessible and systematic way. In this study the authors developed ‘PePApipe’, a custom-built, user-friendly pipeline that enables rapid, complete, and efficient ASFV genome analysis. It is specifically designed for laboratory professionals with limited bioinformatics experience, requiring only basic command-line knowledge. Starting from raw sequencing data, PePApipe integrates thirteen software tools into one automated workflow, covering quality control and pre-processing of raw reads, *de novo* genome assembly and variant calling. Programmed in Python, it can be executed locally through bash scripts, or using a Slurm protocol for batch processing of multiple samples. The main outputs are the ASFV consensus genome sequence and a file listing its putative variants compared to the selected reference genome. PePApipe classifies generated files into structured folders and produces intermediate files that can be used as inputs for further or parallel analyses; users can also enable or disable specific steps in each particular case. This pipeline is adaptable and complementary to downstream steps such as viral genome annotation or genome visualization. By consolidating all stages of viral genome analysis into a single automated workflow, PePApipe reduces the likelihood of user error, and enhances reproducibility and efficiency. This user-friendly pipeline facilitates the transition from sequencing to assembly and downstream analysis of viral genomes, ensuring a fast and reliable response to molecular analysis demands. Finally, the pipeline can be easily adapted to the study of other viral species, expanding its application in infectious diseases surveillance.

## Introduction

Despite the availability of generic bioinformatics tools for genome analysis across a variety of organisms, the intrinsic biological and structural differences between genomes often necessitate the development of specialized tools tailored to the organism of interest—this is particularly true in the case of viruses. One such organism is the African Swine Fever Virus (ASFV), a large, complex, double-stranded DNA virus belonging to the family *Asfarviridae* [1]*.* ASFV is of critical concern to the global swine industry due to its ability to cause a haemorrhagic disease with near 100% mortality in domestic pigs, as well as the substantial trade restrictions imposed on affected regions following confirmed outbreaks.

The absence of an effective vaccine, coupled with the virus’s complex biology and high environmental stability, makes ASFV a formidable challenge for veterinary and agricultural systems worldwide [2,3]. Indeed, ASFV has garnered increasing scientific interest in recent years due to its severe threat to global pig production and food security derived from the worldwide epidemiological scenario. The virus has expanded beyond its original endemic regions in sub-Saharan Africa, initially entering Eastern Europe in 2007 and further spreading throughout Europe, Asia, and reaching the Americas in 2021, which has further intensified the need to understand its pathogenesis, transmission mechanisms, and evolutionary dynamics [4,5]. Moreover, knowing the origin of a new ASFV incursion and tracing the virus spread are critically demanded right after an outbreak occurs in a new region. Furthermore, the socioeconomic impact of ASF outbreaks, particularly in countries with large swine industries such as China or Germany, has underscored the urgent need for more targeted control strategies and improved diagnostic tools [6,7].

The analysis of the ASFV genetic content is key to infer the potential origin of the viral incursion and to inform possible measures that can be put in place to avoid further transmission of the virus, thus enabling a full epidemiological picture of the situation which in turn can help bring the situation back under control.

The problem with viruses in general, and with the ASFV in particular, is that their genome is prone to change [8], and this variability in nature is typically a consequence of its replication *in vivo* when it infects host cells (domestic pigs and wild boars). With a genome of variable size between 170–194 kbp, ASFV is able to exchange large parts of its DNA with itself or with variants of the same virus in the cell environment of the host, with the resulting consequence of producing new genomes. Genome variations can be simply point mutations or small indels (insertions and deletions) occurring not very frequently, but nevertheless altering the original genomes; or these changes can be large indels leading to gene rearrangement, and homologous recombinations could occur as well [9]. An additional peculiarity of ASFV genomes is that both left and right terminal regions of its DNA are very variable compared to the central section; moreover, these terminal regions contain inverted terminal repeats (ITRs), which further complicate both the assembly and annotation of the genomes at the analysis stage [10].

Although several tools exist that can be used to perform assembly and analysis of DNA genomes of viruses (see for example: https://zenodo.org/records/7764938), such as ASFV [11,12], the authors of this work have not found to date a tool able to compile all steps involved in this analysis in a simple and accessible way.

PePApipe is suitable for this purpose and implements the work in a rapid, complete, efficient and clear manner. Moreover, this pipeline has been developed bearing in mind that end-users are mostly laboratory professionals with limited bioinformatics skills.

## Materials and methods

### Design of the algorithm

**Fig 1:**
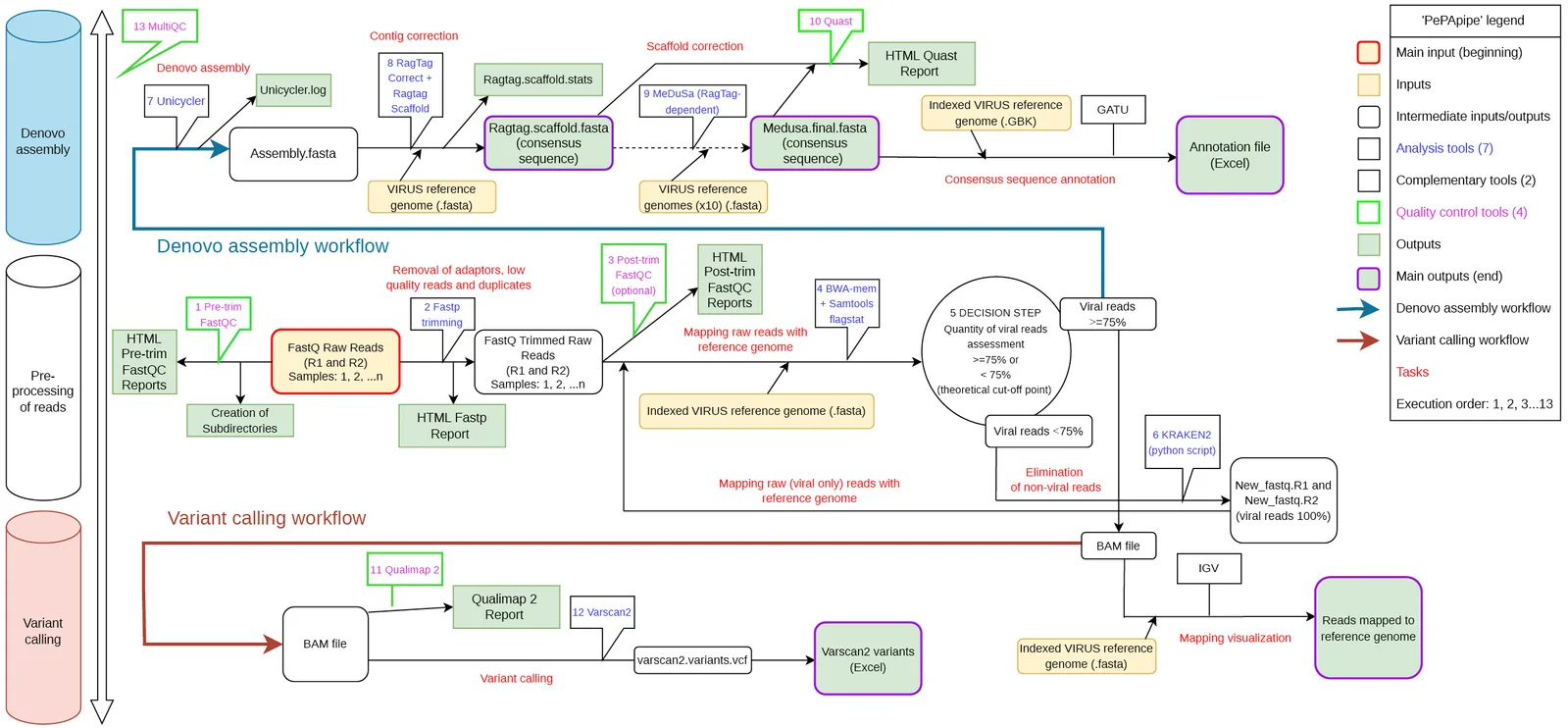
Overall bioinformatics analysis flow followed with PePApipe (in-house built Python pipeline specifically designed for ASF viruses).

This pipeline, developed in Python, can be run using basic Linux commands to launch a Bash script with a Slurm protocol and can be executed on multi-sample batches. The work is sequentially performed over three main areas of genomic analysis: quality control and pre-processing of raw reads, *de novo* genome assembly, and variant calling (Fig 1). Starting from raw data (paired FASTQ files) obtained from short read sequencing platforms (Illumina), PePApipe implements in a sequential manner all crucial steps by using 13 software tools adequately built into a single pipeline.

This pipeline can be run locally or remotely by accessing a computing server. A basic knowledge of Linux programming language is necessary both to install the programmes and to run the pipeline.

The pipeline with all associated information (including the pipeline itself and all accessory scripts) is accessible through the repositories Protocols.io (https://dx.doi.org/10.17504/protocols.io.261geeo57g47/v1) and GitHub (https://github.com/vilocha/PePApipe-pipeline) under the name ‘PePApipe’: a complete bioinformatics analysis pipeline for African Swine Fever Virus genome.

#### Detailed installation steps necessary to build the pipeline.

The following steps must be implemented in the suggested order to successfully install all necessary programmes and be able to run this pipeline. The actual code lines used within the pipeline are clearly shown in the pipeline script itself accessible both through GitHub and Protocols.io. These code lines are already built within the pipeline and will be executed at once when the pipeline is run.

The headers in the bash scripts specify the computation resources needed in the computer or server to allow the execution of the pipeline and its accessories.

According to needs, the different modules in the pipeline can be activated/ deactivated by just uncommenting/ commenting them in the main section of the pipeline script, respectively.

### System and methods implementation

The input or starting point of the pipeline is always the set of raw reads. These raw reads, organized in two files (FASTQ_R1, or forward reads, and FASTQ_R2, or reverse reads) are the standard output of pair-end sequencing protocols when working with Illumina (short reads) or Oxford Nanopore Technologies (ONT) (long reads). The outputs from Pacific Biosciences technology (PacBio) (BAM files) would have to be converted to FASTQ files prior to running the pipeline. The pipeline is structured in a way that it can accept short (Illumina) or long reads (ONT, PacBio) as inputs, although these two last input options have not been tested during the implementation of the study, thus have not been experimentally validated.

The output files of the different steps in this pipeline are specified on the installation webpages accessible through GitHub and Protocols.io. We tested all steps with Illumina sequencing products (paired sampling) from six ASFV samples named E1 to E6 [13].

### Steps

#### Quality control of reads.

This is the initial step which is necessary to ensure a good quality of the FASTQ files provided by the sequencing platform. The quality check of raw reads is carried out with the tool FastQC [14]. The per-base sequence quality is commonly assessed using Phred scores, where values above 28 indicate high-confidence base calls, while scores below 20 are typically associated with increased error rates and are therefore considered suboptimal for downstream analyses [15].

Once the quality control step is complete and one is satisfied that the quality of sequences is adequate to continue the processing, the reads are ‘cleaned’ with the tool fastp [16]. This step is necessary to ensure that adapters and low quality/ duplicate reads are removed off the FASTQ files. In addition, fastp produces a post-trimming quality analysis report, making it unnecessary to perform a further stand-alone quality control with FastQC.

Only reads with a minimum Phred quality score of 20 (the default fastp Phred quality score is 15) and a minimum length of 15 base pairs (default parameter) are kept after applying fastp, but these parameters can be changed in the code line according to user needs. Depending on the number of reads available, these parameters can be changed up or down with the aim of maximizing output and minimizing low quality reads.

A minimum average sequencing depth of ≥30x and a genome coverage breadth of at least 90–95% were considered necessary for successful genome assembly using this pipeline. The depth threshold of 30x was defined based on empirical evaluation, as samples below this value showed reduced assembly completeness and samples above this value did not contribute to substantially increase assembly completeness. The minimum coverages obtained at different depths were 91.40%, 98.30% and 98.40% at 20x, 30x and 40x, respectively. Moreover, a minimum mean sequencing depth of 30x is indicated on published recommendations to result in high-quality viral consensus genomes. Genome coverage of at least 90–95% was selected to ensure that reconstructed ASFV genomes were sufficiently complete for downstream analyses such as comparative genomics and phylogenetic inferences [17].

#### Viral reads content check.

In this step the reads that passed the previous filters (‘clean reads’) are mapped against the virus reference genome (in FASTA format) chosen for convenience in each particular case using the software tools BWA-mem2 [18] and Samtools [19].

This step is probably the most important of all steps included in this part of the flow, and of the pipeline for that matter. The viral genome reference used for convenience by the authors of the pipeline was ASFV Georgia 2007/1 (Genbank assembly: GCA_003047755.2), since it was the initial isolate of genotype II emerging in Eastern Europe in 2007 and is considered by the scientific community as the reference strain.

The objective of this mapping is two-fold: on the one hand, it allows to quantify the amount of reads within the original FASTQ files actually mapping with an ASFV (and thus to also quantify the amount of reads not mapping with an ASFV), and on the other hand, it creates a set of reference-mapped contigs (BAM file) as a first step in the search of genetic variants (SNPs and indels) in the genome of our virus problem compared to the genome of the virus reference used.

#### Quality check of reads mapped to reference genome of choice.

One of the tools most commonly used for this purpose is Qualimap 2 [20]. The rationale behind is to assess the quality of the mapping of trimmed raw reads to the virus reference genome (performed before to calculate the amount of viral reads present). The outputs can be verified in PDF and HTML formats.

#### Quantitative assessment of viral reads (theoretical cut-off point: 75%).

It is important to carry out all bioinformatics analyses only on viral reads free of contamination with reads from other origins (host, environment or handling personnel) or, if this is not possible, at least on a number of viral reads as high as possible. This is because the results we obtain may be spurious if the analyses are performed on a high number of non-viral reads, and therefore not representative of the virus we are trying to characterize.

This is a point in the pipeline where a decision has to be made as to the quality level we want to apply to our analysis in relation to the available reads (Fig 1). A theoretical threshold (cut-off point) of 75% of viral reads was established to determine whether to proceed with the already filtered FASTQ files (‘clean reads’) or to perform an additional filtering step to remove non-viral reads. This threshold was chosen based on preliminary analyses, which indicated that samples with lower proportions of viral reads exhibited increased host contamination and reduced reliability in downstream analyses, and on previous studies highlighting the impact of non-target reads on metagenomic classification accuracy and downstream processing [21,22]. The viral read threshold was therefore selected as a conservative default quality indicator reflecting sample enrichment rather than a universally accepted quality standard (in our study the lowest percentage of viral reads mapped to the reference genome was 76.10%). Because the proportion of viral reads depends on sample type and sequencing protocol, this parameter is configurable by the pipeline user.

The quality control thresholds implemented in PePApipe are intended to facilitate reliable ASFV consensus genome reconstruction. They are not intended as universally optimal cut-offs and can be modified by the user according to sequencing protocol, sample quality and analytical objectives.

Apart from the methods used to isolate the viral DNA, the method used to prepare the libraries (with or without enrichment) is the single most influential step on the amount of foreign (non-viral) DNA reads found in our sample. If a non-targeted or non-enriched library preparation method is used, there will be a large number of non-viral reads present in the FASTQ files, thus these may be larger in size although not necessarily better in quality.

Hence, this decision threshold is arbitrary and may vary depending on the initial FASTQ files and whether the library has been prepared using enrichment or not. Libraries prepared for shotgun sequencing are likely to contain <1% of viral fragments.

#### Removal of reads not belonging to viruses.

If we decide to carry out another filtering step to remove non-viral reads, a new set of FASTQ files must be created by mapping the ‘clean reads’ against all published reliable reads belonging to the Superkingdom Viruses (NCBI: txid10239). To carry out the removal of non-viral reads a loop with an additional Python script using the tool Kraken2 [21] is necessary. The procedure consists of two steps: building of the virus database and extraction of the viral reads. This extra filtering step will create a new set of FASTQ files containing only viral reads.

As mentioned above, this procedure can be performed whenever the chosen threshold of viral reads is not reached (in our case approximately 75%) or it may also be systematically run every time, independently of the amount of non-viral reads present in the original ‘cleaned’ set of FASTQ files.

If there is availability of a substantial amount of samples, a benchmark exercise can be carried out to refine the decision threshold or cut-off percentage point, making it lower or higher than 75% according to each specific situation.

#### *De novo* viral assembly.

This step is mainly performed with the software tool Unicycler and ultimately refined with the programmes RagTag (using Unicycler outputs as inputs) and MeDuSa (using RagTag outputs as inputs).

These tools use several dependency programmes in order to obtain a full viral *de novo* genome assembly starting from the FASTQ files containing our already ‘cleaned’ reads, be these the original FASTQ files or the new set created using Kraken2.

Although Unicycler [23] was originally built for bacteria, ideally combining both short (Illumina) and long reads (ONT, PacBio), it performs equally well using only short reads from viral genomes.

A most positive aspect about this type of assembly is that a viral reference genome is not used at all, thus no bias from this source can be introduced, allowing for a genuine assembly of the putative whole genome of our virus problem. This step is critical within the *de novo* assembly part of the flow, but unfortunately it is also the most consuming in terms of time and resources.

There are particularities in relation to the three tools used in the *de novo* assembly. In order to obtain a proper assembly of our virus's whole genome it is important to specify that what we are looking for is a linear sequence, in other words, that the expected number of linear (non-circular) sequences in the underlying sequence being assembled is just one, and so these requirements must be included in the code in the Unicycler section.

After the first assembly step with Unicycler is concluded, a correction of the obtained contigs converting these into scaffolds is performed with the tool RagTag [24]. This step is necessary to guide the *de novo* assembly performed by Unicycler into plausible contigs belonging to the ASFV, using for that purpose the chosen viral reference genome in FASTA format (in our case ASFV Georgia 2007/1; Genbank assembly: GCA_003047755.2).

It is important to note that, even though a viral reference is used, not a single fragment from it is incorporated into the assembly obtained with Unicycler, so the only activity performed in this step is contig correction and scaffolding. RagTag comprises four software tools (‘correct’, ‘scaffold’, ‘patch’ and ‘merge’) but only the first two are used in the pipeline. ‘Patch’ is not used to avoid adding viral reference fragments and ‘merge’ is not necessary since only ‘scaffold’ was used as scaffolding method.

After the correction and scaffolding performed by RagTag, a third tool called MeDuSa [25] is incorporated as part of the *de novo* assembly process. MeDuSa performs a scaffold correction in a similar way to RagTag, but instead of using only one viral reference it uses as many well curated references as we are able to provide, always making sure the references are akin to the ASFV genotype we are studying. References belonging to genotype I should be used when working with this genotype. Up to 10 references belonging to ASFV virus genotype II have been included in this pipeline, which in alphabetical order are: Arm_07_CBM_c2_LR812933.1, ASFV_Georgia_2007_1_FR682468.2.fasta, ASFV_HU_2018_MN715134.1, ASFV_LT14_1490_MK628478.fasta, Belgium_2018_1_LR536725.1, Estonia_2014_LS478113.1, Korea_YC1_2019_ON075797.1, POL_2015_Podlaskie_MH681419.1, Tanzania_Rukwa_2017_1_LR813622.1 and Ulyanovsk_19_WB_5699_MW306192.1.

The higher the number of references used, the better the result of this step will be. Briefly, the contigs obtained from the previous step are further assembled into larger scaffolds with MeDuSa. If the quality of the original reads is high this step generally results in one final contig, which is the putative consensus sequence of the virus under study. On the contrary, poor quality sequences may result in a large number of contigs, and if the number of contigs exceeds 3–5 it would be advisable to reject those sequences.

#### Quality assessment of the *de novo* assembly.

This is a quality analysis that can be performed over all steps in the *de novo* assembly loop with the intention of investigating the overall quality of the process. The tool included in the pipeline for this task is QUAST (QUality ASsessment Tool) [26] and the outputs are available on different formats: TXT, PDF, TSV, HTML, etc.

#### Search for viral genome variants (variant calling).

Several tools are used to reveal genetic variants (SNPs and indels) present in the virus genome under study when compared to a viral reference genome.

In general, there are two main types of tools: those that use a Bayesian inference and those that use a deterministic/numerical approach. The tool included in this pipeline is called VarScan 2 [27] and is one of the second type (numerical approach).

Variant calling was performed with VarScan 2 [27], using conservative thresholds selected to reduce false-positive calls: a minimum of 5 reads supporting the variant, a minimum coverage depth of 20, a p-value threshold of 0.001, a minimum average base quality of 30, and a minimum variant frequency for each position of 0.05 (to account for true minority variants). These parameters are more stringent than the default VarScan 2 settings and were chosen to increase the reliability of variant detection in our pipeline. Previous studies showed that higher coverage and more restrictive read-support and statistical thresholds improve call confidence [27–29].

We used a tool for variant calling provided by Qiagen CLC Genomics Workbench [30] to compare the variant calling results obtained with VarScan 2 to ensure that the latter performs adequately.

#### Overall quality assessment of the analysis.

In order to perform an overall assessment of the analysis, the tool MultiQC [31] may be used. This tool evaluates all inputs and outputs in the working directory and performs a general quality summary assessment, which is based on the quality of the inputs (FASTQ files) and on the type of programmes used. This tool must be executed in the working directory where all folders used during the running of the pipeline are located. This final step is valuable if we want to perform benchmark exercises to compare different inputs or different tools or both.

#### Expected outputs and interpretation.

The pipeline produces all necessary files to interpret the results, classifying them into folders. Processing times depend on the size of the FASTQ files, from 10 minutes with files of around 0.5 Mb through to 30 minutes with files of 1 Mb in size, or to longer times if high capacity sequencers are used. Several intermediate files are also created in the process, which can be used as inputs for further or parallel analyses, as well as to check for possible errors during the analysis. Among these complementary post-analysis steps are viral annotation with GATU (https://4virology.net/virology-ca-tools/gatu/) or genome visualization with IGV (https://igv.org/).

All steps are amenable to user control by means of switching on/off (uncommenting/ commenting, respectively) the necessary sections in each particular case. There are several steps where the quality of the processes can be checked to make sure the progress of the results is in line with the user expectations.

The pipeline can be easily adapted to viruses other than ASFV by changing the parameters relevant to the new virus and running the specific pipeline sections accordingly.

The most important outputs after running the pipeline are the consensus sequence of the genome of the virus we are studying and the table of variants for that particular virus against the chosen reference genome. Secondary outputs are: reference mapping parameters, *de novo* assembly parameters, and quality assessment summaries.

PePApipe is user-friendly and can be installed through a straightforward series of steps. While it is primarily designed for analyses involving short reads, it is also compatible with long reads. The outputs are consistently organized into folders, providing a structured basis for further analyses. Additionally, the code is open-source and can be modified and adapted to meet user specific needs, provided that appropriate credit is given.

## Results and discussion

The pipeline described on this paper has been built having into account the specific characteristics of ASF viruses. Every step was tried on the sequences derived from the six samples operating multiple tools commonly used for that purpose. After different runs, the results using different tools were compared with the rest and the best among them were used to decide upon which tool to include in the pipeline.

To examine the quality of raw reads we included FastQC [14] only once for an initial quality assessment and used fastp [16] instead for the post-trimming quality assessment. This is because fastp includes this assessment as a subsequent step to the trimming process. To decide on a trimming tool we considered trimmomatic [32], cuatadapt [33], and fastp [16], and we selected the latter based on the just mentioned post-trimming quality check it provides.

We tested the trimming with the three tools mentioned above, applying a common minimum length for trimmed reads of 60 and two minimum quality thresholds: 20 and 30. The reads obtained with quality thresholds of 20 were consistently longer than the reads obtained when we were stricter with the quality threshold (30), hence we opted to include quality thresholds of 20 in the trimming tool. In our experience, when working with viruses, unless the analyst has an exceptionally good sequencing sample, it is better not to be too strict on quality of reads. And this is particularly true when the amount of viral DNA at the outset of the experiment is not too high, even after using viral enrichment methods to prepare specific libraries prior to sequencing, as it was our case.

For the *de novo* assembly stage we focused on the tool Unicycler [23] which has been shown to perform well on assembly of viruses. Moreover, although it is a tool designed to combine short and long reads to perform a hybrid assembly, it delivers similar results with viruses working with short reads only, which is an advantage in this case. Of course, the tool is equally capable of analysing a mixed of viral short and long reads. Unicycler already includes other assembly and polishing tools, such as SPAdes [34] and Pilon [35].

Two different software programmes were tested for *de novo* assembly of the sequences: SPAdes and Unicycler (Table 1). The Unicycler software was used with the parameter --linear_seqs 1, forcing it to return a single contig. We observed a significant difference in the number of contigs corresponding to ASFV obtained with each programme. The number of contigs obtained with SPAdes ranged between 16 and 504, whereas with Unicycler was between 3 and 6, demonstrating the extra scaffolding gained with other programmes included in Unicycler such as Pilon [35].

**Table 1:** Comparison of parameters obtained using SPAdes and Unicycler as *de novo* assembly tools for ASFV sequences.

SAMPLE	ASFv contigs SPAdes	ASFv contigs Unicycler	Longest contig SPAdes	Longest contig Unicycler
E1	16	3	185354	185352
E2	120	3	185506	185352
E3	120	4	115053	185352
E4	256	6	113089	185352
E5	504	3	30747	125185
E6	117	4	145211	185353

It is worth noting that we obtained more contigs with SPAdes than those shown in Table 1, but we have only displayed the contigs corresponding to the virus of interest. In Sample E2, for example, nearly 9000 contigs were obtained that did not correspond to ASFV, but to other organisms. This large number of contigs obtained with SPAdes complicates the analysis of the virus that is of interest to us.

Differences in the length of the longest contig were also observed. With Unicycler, we obtained a contig between 40k and 90k nucleotides longer in 4 out of the 6 samples. In the samples where we obtained shorter contigs with Unicycler, the difference ranged from 2 to 154 nucleotides, therefore we chose Unicycler as the best *de novo* assembling tool for the virus of interest.

One of the main challenges when performing bioinformatics analyses of viral sequences is the need to obtain the truest and most accurate consensus sequence of the virus being analyzed. Any rough approximation is useless and we must aim to obtain the true genetic picture of the virus we are confronting with. To attain this goal, we included a combination of two published and reliable analysis tools in our pipeline: RagTag and MeDuSa.

Two of the four available functionalities from RagTag [24] are used in the pipeline: ‘correct’ and ‘scaffold’. Most of the contigs are joined together using a virus reference genome as a guide but without inclusion of any of the nucleotides from the viral reference sequence. Once this compilation job is completed, MeDuSa [25] implements a final join of the remaining contigs, this time using as references as many as we can/want to provide. In our case we used 10 references of ASFV genotype II, as these were phylogenetically closest to our problem viruses. If working with genotype I, references akin to this genotype should be used instead.

MeDuSa attempts at further connecting contigs by matching our problem virus with the sequences of the references provided; and similarly to RagTag, it does not use any of the nucleotides off the references themselves.

In our experience, approximately 80% of the assemblies we obtained were reduced to a single contig, and in the remaining 20% of cases the final contig sets included up to three and, in very few occasions, up to five contigs. The two main reasons for not being able to assemble to a final single contig were either lower quality of reads or possible contamination (in our case with bacterial plasmids of around 5,000 nucleotides long). Typically, we accepted as good assemblies consensus sequences consisting of a single contig.

Assembly quality was assessed using QUAST [26], considering key metrics including total assembly length, number of contigs, N50, genome coverage, and GC content. Assemblies were considered acceptable when they met the following criteria: genome coverage ≥90–95%, low fragmentation (reduced number of contigs), N50 values consistent with expected genome size, and absence of major deviations in GC content compared to reference genomes. Assemblies without these criteria were considered suboptimal and subjected to further inspection or reprocessing.

For the alignment with reference (mapping) of our sequences we tested mainly two viral references: ASFV Georgia 2007/1 (GenBank assembly GCA_003047755.2) and ASFV Lithuania LT14/1490 (GenBank assembly GCA_008932025.1). Although all results were very similar in both cases in terms of quality and metrics, we opted for ASFV Georgia 2007/1 for all the analyses, as it represents the initial reference strain of the entrance of ASFV genotype II into the Eurasian territory. For alignment of genotype I viruses an appropriate genotype I virus reference should be used.

**Fig 2:**
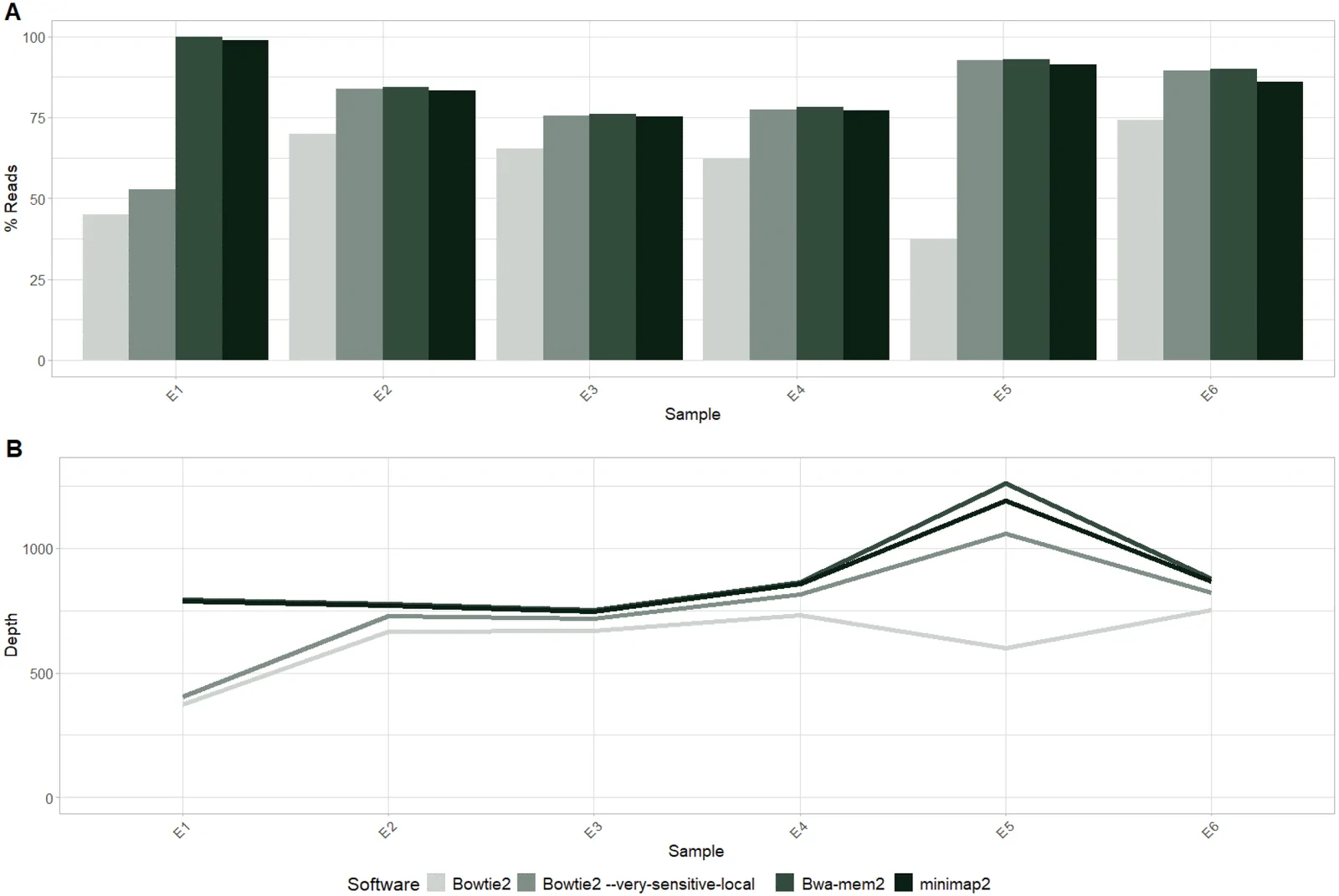
Comparison of Bowtie2, bwa-mem2 and minimap2 as mapping tools for ASFV sequences.

For the mapping, three different software programmes were tested: minimap2 [36], Bowtie2 [37] and bwa-mem2 [18] (Fig 2). Bowtie2 was used with two different parameter sets: --very-sensitive-local and the default parameters. Both minimap2 and bwa-mem2 perform local alignment by default, meaning that the entire read does not need to match the reference genome. Bowtie2 does not use this type of alignment by default, so the corresponding parameter must be added manually.

The percentage of reads mapping to the reference was higher in all cases with the bwa-mem2 software. As expected, the lowest percentages were obtained with Bowtie2 using the default parameters, although in five out of the six samples analyzed, Bowtie2 with the local mapping parameter was the second software that mapped most reads (Fig 2). The bwa-mem2 software was ultimately selected because it achieved higher percentages of mapped reads in all samples, optimizing the variant calling process. If using long reads, minimap2 mapper should be used as it increases speed and accuracy of alignment.

This pipeline can process recombinant viruses (as it is the case of ASFV recombinants of ASFV genotype I and genotype II circulating in China) but it is not able to claim recombined areas as such. One strategic solution would be to map everything against genotype I, map everything against genotype II, and then look out for regions where mapping quality changes between references in both mapping outputs. These regions could represent the cross-over points between recombinant fragments. In these cases, instead of a single consensus sequence, several contigs may be obtained at the *de novo* analysis stage, as it happens with poor quality alignments. But it will not be possible to ascertain if the cause of these alignments is low quality initial inputs or the result of recombined viruses.

This pipeline yields optimal results when used in conjunction with an enrichment strategy designed to isolate and characterize viral sequences, such as through a custom-built viral genome library or by using an amplicon-based approach. In the absence of such capture step, the proportion of non-viral reads is likely to be very high, which can significantly reduce the reliability of the results compared to those obtained with prior enrichment.

Table 2 shows, as an example, a summary of results and statistics of complete genome analyses from the six different ASFV viral samples included in this study (in columns). The different metrics shown (in rows) give an idea of the variability observed among these viral samples.

**Table 2:** Example of summary of results and statistics of complete genome analyses of several viral strains.

Statistics	Sample E1	Sample E2	Sample E3	Sample E4	Sample E5	Sample E6
Per base sequence quality (average R1 + R2)	36	36	36	36	36	36
% G + C (average R1 + R2)	39%	38.5%	39%	39%	41%	38%
Total number of reads	1,049,376	1,211,198	1,298,236	1,486,248	2,055,450	1,282,808
Duplication rate	33.9%	17.4%	18.7%	22.3%	18.5%	15.7%
Reads passing quality filters	1,038,682	1,198,676	1,283,168	1,461,022	2,039,472	1,268,262
% reads passing quality filters	99.98%	98.97%	98.84%	98.30%	99.22%	98.87%
Reads mapped to Reference genome	1,036,938	1,009,847	976,483	1,142,610	1,897,109	1,141,496
% reads mapped to Reference genome	99.83%	84.25%	76.10%	78.21%	93.02%	90%
Mean coverage per contig (depth or vertical coverage)	795	777	751	863	1,260	877
No. of initial fragments before MeDuSa step at *de novo* assembly	2	3	2	3	5	2
No. of final fragments after MeDuSa step at *de novo* assembly	1	1	1	1	1	1
Total *de novo* assembled length (horizontal coverage)	187,794	187,892	187,891	187,898	188,341	187,893

The analysis of variants in genomic sequences that are subject to high variation, such as those obtained with viruses, is not an easy task. ASFV contains a DNA genome and is expected to show a low level of mutation. However, its large size (up to 194 kbp) and its ability to delete, insert, duplicate, invert, and recombine short and large genome regions, make variant calling a challenging step. We explored several tools to try to standardize this search. Initially we used the Nextflow platform-based [38] nf-core/viralrecon, which is a tool that was developed during the Covid-19 pandemic to analyze new coronavirus variants [39]. We found its installation and processing cumbersome, and the outputs did not fulfil the expectations we had beforehand about this tool.

After searching for other appropriate tools we decided to compare two bayesian inference searching tools (BCFtools [19,40] and Freebayes [41]) with a non-bayesian tool (VarScan 2) [27], using an additional fourth tool (basic variant detection included in the CLC Genomics Workbench software) [30] to analyze the overall results from four perspectives, performing in this way an orthogonal analysis. The main finding after using the four tools was the lack of variants found by the two Bayesian methods, compared to VarScan 2 which found several variants. Overall, CLC found the same variants as VarScan 2, and the minimal discrepancies between the two were mostly related to considering or not considering non-canonical nucleotides as variants.

In our opinion, and considering the viral variability, it was unexpected to find that the viral sequences we analyzed did not have any variants with regards to the references used. Therefore, BCFtools or Freebayes could be more adequate for other types of genomes, and we selected VarScan 2 as the main tool for variant calling.

The validation of variants provided by VarScan 2 was carried out using a visualization tool [IGV (Integrative Genomics Viewer)] [42–45], which is the software tool most universally used for visualizing genome mappings. This mapping is an optional step and is not an integral part of the pipeline.

The *de novo* consensus sequence built by the pipeline can be annotated using a reference genome of choice. Although this is neither an essential step in the pipeline but rather a complementary analysis that can be carried out alongside, it is an essential task to identify and tag the different genes that have been assembled together. A popular tool released so far for this purpose is a universal tool for general annotation that works well with viruses (and specifically with ASFV, although it is not fully fit for annotating this virus) called GATU (General Annotation Transfer Utility) [46].

To our knowledge, two other pipelines have been described to date to specifically analyze ASFV genome reads, revealing the necessity and relevance of ASFV tailored bioinformatics solutions. The first, published by Spinard *et al.* in August 2024 [11], is designed to process both short and long reads. The second, ANASFV [12], developed by Li *et al.* and released in 2025, is specifically tailored for long reads.

The pipeline developed by Spinard *et al.* was tested on two genomes only, while PePApipe was evaluated with six genomes. Although expanding the number of samples for testing would be ideal, this is not always feasible. Nevertheless, additional samples can be obtained from public repositories to increase the dataset size. However, the sequencing quality or the total viral read count from such sources may vary and not always meet desired standards.

For short read analysis, Spinard *et al.* employed a reference-based approach which, as mentioned earlier, can introduce identification bias by limiting the discovery of novel genomes. On the other hand, their use of *de novo* assembly with both short and long reads helps resolve or better elucidate ITRs compared to short reads alone. To address this limitation, PePApipe also supports a combined approach using short and long reads, with long reads incorporated in the initial *de novo* assembly step using Unicycler. One important difference between the pipeline by Spinard *et al.* and PePApipe is that the former allows submission of up to four datasets for analysis, whereas PePApipe can process as many samples as needed in batch mode, albeit at the expense of longer processing times.

The minimum number of samples to process in each run with PePApipe should be 1 (2 FASTQ files) and ideally a maximum of 10 samples (20 FASTQ files). We carried out the analyses with FASTQ files ranging from 500 Mb to 1000 Mb each (Table 2), but the pipeline can accommodate FASTQ files of 1200 Mb or higher (in the latter case at the expense of longer processing times).

In our analyses, more than 50% of Illumina reads mapped to both the ASFV virus and *Sus scrofa* (host) genomes. The straight removal of all reads mapping to *Sus scrofa* would mean losing as well an important amount of reads mapping to the ASFV virus, which are essential for the viral genome assembly. Moreover, the pipeline by Spinard *et al.* does not use reads mapping to both the ASFV virus and host to find the most similar reference to their genome (among 33 reference genomes). This may result in an inadequate identification of such reference genome. Although 10 reference genomes were used in PePApipe (through the use of MeDuSa), a larger panel of reference sequences can be used. The final consensus sequence may be more reliable at the expense of prolonging processing times, although the assembly improvement is likely to be marginal.

The pipeline by Spinard *et al.* uses the *de novo* assembled contigs to predict a closely related reference genome whereas PePApipe do not make inferences about a reference genome. Instead, it constructs the *de novo* assembly in one go by combining the use of RagTag and MeDuSa.

Moreover, Spinard *et al.* use reference genomes to align ITRs segments that remain unaligned and flanking those ITRs segments which their pipeline could align in the first round. This practice can curtail the possibilities of correct alignment of open reading frames (ORFs) not present in such reference genomes but present in a potential ‘new’ virus.

The search for variants strategy of Spinard *et al.* is based on the use of BCFtools. We hardly found variants using this tool, as explained above. The reason for this difference could be that we used samples that were very similar between each other, and between them and the sequence of the reference genome, since they were all ‘in vivo’ passes of the same virus. Using BCFtools with a more heterogeneous dataset may result in more variants being called.

The publication describing the ANASFV pipeline does not provide as detailed an explanation of the assembly stage as PePApipe or the pipeline by Spinard *et al.* ANASFV employs reference genomes to correct low quality reads typical of ONT sequencing, which guides the assembly process but may overlook ORFs not present in the reference genomes. Additionally, the authors highlight that substantial host DNA contamination is a significant challenge in ONT sequencing, often leading to inadequate coverage. Rather than completely removing all reads that map to the host (*Sus scrofa*), this problem might be better addressed by removing only those reads that map exclusively to the host but not to the ASFV genome.

Low-coverage regions are not explicitly treated in our pipeline; instead, these are indirectly filtered out through variant calling thresholds and rejection of low quality assemblies. Poor quality FASTQ inputs may result in missing or unreliable sequence information in poorly covered genomic regions.

Repetitive regions and multigene families (MGFs) remain a challenge. These regions may lead to fragmented assemblies, collapsed repeats, or misassembled scaffolds, especially at the variable terminal regions of the genome. This is an inherent limitation of assembly approaches using only short reads, hence, the incorporation of long reads within the pipeline can help mitigate some of these issues by spanning repetitive regions. Future developments of PePApipe will aim to further improve the resolution of these complex genomic regions.

In PePApipe, ITRs regions are not addressed through a dedicated or specialized module. Instead, they are handled implicitly within the general *de novo* assembly and scaffolding framework. The pipeline uses Unicycler for initial assembly, followed by scaffolding and correction steps with RagTag and MeDuSa. This combined approach improves contiguity and allows partial reconstruction of terminal regions if there is sufficient coverage and sequence continuity.

However, due to the repetitive nature and structural complexity of ITRs, short read assemblies may not fully resolve these regions. This can result, as mentioned above, in fragmented assemblies and collapsed repeats, or in uncertainty in the exact structure and length of terminal sequences. The use of multiple reference genomes in the scaffolding stage may help guide contig orientation in these regions, but it does not fully overcome the inherent ambiguity of repetitive sequences. As pointed above, our pipeline allows the incorporation of long reads, which can span repetitive regions, and therefore has the potential to improve the resolution of ITRs. In fact, combining a short and long reads sequencing approach is currently the most effective strategy to overcome this challenge, although this may incur in an increase in lab reagents, equipment, resources, and labour investment.

This study has several limitations that should be acknowledged. First, although PePApipe supports both short and long reads, the current validation was performed using only short reads, lacking experimental validation of hybrid or long read assembly workflows. Second, the comparison with other ASFV-specific bioinformatics pipelines was not based on a direct benchmarking framework, and neither used identical datasets, which may affect the comparability of results. Third, this study considered a limited validation dataset consisting of six samples, thus, the obtained results must be interpreted in this context. Fourth, since PePApipe has been experimentally tested with short reads, the resolution of repetitive genomic regions, MGFs or ITRs remains challenging, and instead are subjected to inaccurate or fragmented assemblies. Finally, PePApipe does not include dedicated modules for recombination detection or explicit handling of low-coverage regions, which may impact the interpretation of complex genomic features, therefore it is not designed as a recombination-detection tool and cannot reliably identify recombination breakpoints or cross-over events without additional specialized analyses.

Future work will aim to address these limitations by expanding benchmarking datasets, incorporating long read sequencing validation and integrating additional analytical modules, such as specific genome annotation better tailored to ASFV.

A structured comparison of PePApipe with the other ASFV-specific pipelines is provided in Table 3, highlighting differences in assembly strategy, input data requirements and performance metrics.

**Table 3:** Structured comparison of PePApipe with other ASFV-specific pipelines.

Feature	PePApipe	Spinard *et al.*, 2024 [11]	Li *et al.*, 2025 (ANASFV) [12]
Input data	Short + long reads	Short + long reads	Primarily long reads
Assembly strategy	Double (*de novo* + reference-guided)	Hybrid (*de novo* + reference-guided)	Reference-guided correction + assembly
Reference usage	Used for scaffolding only (no sequence incorporation)	Used for reference selection and alignment	Used to correct reads and guide assembly
Variant calling	VarScan 2	BCFtools	Not clearly specified/ limited detail
Samples tested	6 genomes	2 genomes	Not clearly specified
Handling of host reads	Removes host reads	Removes host reads	Highlights host contamination as major issue
Batch processing	Yes (Slurm support, scalable)	Limited (up to four datasets)	Not clearly specified
Output	Consensus genome + variant table + QC metrics	Assembly + annotation	Assembly + evaluation
Repetitive regions/ MGFs	Improved with long reads (not experimentally validated)	Improved with long reads	May be biased by reference-guided approach
ITRs handling	Implicit via assembly/scaffolding	Uses reference-guided alignment of ITRs	Limited detail provided
Strengths	Integrated, user-friendly, scalable, *de novo* approach	Hybrid assembly improves complex regions	Optimized for long read data
Limitations	Assembly of repeats, ITRs and recombination	Potential reference bias	Possible loss of novel regions due to reference guidance

Regardless of the pipeline used, a suitable computational infrastructure and personnel with basic bioinformatics expertise are essential. However, we believe PePApipe may need lower computer resources than other pipelines as it was designed with the aim of providing simplicity in the first place. Every step is clearly detailed, as can be found in the pipeline access websites.

## Conclusions

In this study we present ‘PePApipe’, an automated and user-friendly computational pipeline designed to simplify and standardize the complete genomic analysis of ASFV. Starting directly from raw sequencing data, PePApipe guides users through all essential steps, from quality control to genome assembly and variant detection, producing reliable and reproducible results in a short time.

By integrating multiple established tools into a single workflow, and providing clear intermediate outputs, our approach reduces the risk of user error and increases transparency. Importantly, we designed PePApipe with accessibility in mind, enabling laboratory scientists to perform advanced genomic analyses with minimal computational background. While developed for the ASFV, the pipeline can be adapted to other viruses, making it a flexible resource for viral genomics, outbreak investigation, and future data-driven research.

The future plan is to include a viral genome annotation tool in the pipeline to harmonize this crucial and complex step, especially for a large virus such as ASFV, which can encompass more than 180 ORFs along the genome.
